# Tunnel technique with enamel matrix derivative in addition to subepithelial connective tissue graft compared with connective tissue graft alone for the treatment of multiple gingival recessions: a randomized clinical trial

**DOI:** 10.1007/s00784-020-03312-6

**Published:** 2020-05-07

**Authors:** Bartłomiej Górski, Renata Górska, Joanna Wysokińska-Miszczuk, Tomasz Kaczyński

**Affiliations:** 1 Department of Periodontology and Oral Mucosa Diseases, Medical University of Warsaw, S. Binieckiego 6 St, 02-097 Warsaw, Poland; 2 The Chair and Department of Periodontology, Medical University of Lublin, Karmelicka 7 St, 20-081 Lublin, Poland

**Keywords:** Enamel matrix derivative, Esthetics, Modified coronally advanced tunnel technique, Multiple gingival recessions, Subepithelial connective tissue graft

## Abstract

**Objectives:**

The aim of this study was to compare outcomes of the modified coronally advanced tunnel technique (MCAT) combined with subepithelial connective tissue graft (SCTG) with or without enamel matrix derivative (EMD), in the treatment of gingival recession types 1 and 2.

**Materials and methods:**

A total of 20 patients with 150 multiple gingival recessions (GR) were included in the study. On one side, MCAT was combined with SCTG and EMD (tests), whereas MCAT with SCTG was applied on the contralateral side (controls). Clinical parameters were measured at baseline and 6 months after surgery. Visual analog scales (VAS) and questionnaires were used to assess patient-reported outcomes and the root coverage esthetic score (RES) for professional esthetic evaluation.

**Results:**

MCAT+SCTG+EMD was not superior with regard to root coverage. At 6 months, average root coverage (ARC) was 87.4% for SCTG+EMD-treated and 90.9% for SCTG-treated defects (*p* = 0.4170). Complete root coverage (CRC) was observed in 86.7% (tests) and 85.3% (controls) of the cases (*p* = 0.9872). Significantly less pain was reported using VAS (*p* = 0.0342) post-operatively in the SCTG+EMD group. Professional assessment of esthetic outcomes using RES showed a significant difference (9.25 versus 8.71, *p* = 0.0103) in favor of the test group.

**Conclusions:**

Both treatment modalities were equally effective in treatment of multiple GR and led to similar improvements in clinical parameters. However, the application of EMD as an adjunct resulted in less post-operative pain and better professionally assessed esthetic outcomes.

**Clinical relevance:**

Patients’ early morbidity and 6-month esthetic outcomes following GR coverage with MCAT might be influenced by means of EMD utilization.

## Introduction

Gingival recession (GR) is described as an apical shift of the gingival margin that is associated with clinical attachment loss (CAL). Its frequency increases with age, as 50% of people aged 18 to 64 years and 88% of people older than 65 years have at least one GR [1]. It may be caused by different conditions, such as plaque-induced inflammation, improper toothbrushing, intrasulcular restorative/prosthetic cervical margin placement, periodontal disease, and orthodontic treatment [2]. Among possible consequences of root surface exposure to oral environment besides impaired esthetics are dentin hypersensitivity, caries and non-carious cervical lesions. A modern treatment-oriented classification of gingival recession based on interdental CAL evaluation was proposed by Cairo et al. [3]. This system distinguishes between three types of defects: (1) recession type 1 (RT1) with no loss of interproximal attachment, (2) recession type 2 (RT2) when the amount of interproximal attachment loss is less than buccal attachment loss, and (3) recession type 3 (RT3) in case of interproximal attachment loss greater than the buccal attachment loss. This classification overcomes some drawbacks of the widely used Miller classification [4]. By and large, RT1 encompasses Miller classes I and II, RT2 overlaps Miller class III, whereas RT3 overlaps Miller class IV.

A wide range of surgical treatment modalities for correcting GR have been developed. Among many different approaches, the tunnel technique, which was first described by Zabalegui et al. [5], has lately gained popularity. However, the efficacy of the tunnel technique was dependent on the application of subepithelial connective tissue graft (SCTG) [6]. Owing to limited flap opening and elimination of vertical cuts, this treatment strategy provided greater blood supply and graft nutrition, faster healing, and reduced post-operative morbidity [7]. Recent systematic review and meta-analysis showed that the tunnel technique is a highly effective procedure in treating multiple GR [8]. Ever since the introduction of the original technique, further modifications with the aim to enhance final clinical outcomes have been proposed over the years by several researchers [9–11].

Recommendations were made for an addition of enamel matrix derivative (EMD) as an adjunct to soft-tissue grafting to improve treatment efficacy [10, 12]. EMD was applied for root coverage to improve the level of gingival margin and enhance periodontal regeneration along the root, which was confirmed with histologic analysis. It was shown that application of EMD resulted in re-formation of root cementum, periodontal ligament, and alveolar bone, whereas treatment with SCTG led to long junctional epithelium establishment and even root resorption [13, 14]. Moreover, EMD was found to play a key role in wound healing-promoting angiogenesis, revascularization, and soft-tissue regeneration [15], as well as enhanced collagen synthesis and the expression of transforming growth factor (TGF) β1 and TGF β2, vascular endothelial growth factor (vEGF), interleukin (IL)-1β, matrix metalloproteinase (MMP)-1, versican, and fibronectin [16]. Quite recently, Shirakata et al. [17] reported significantly higher improvements in clinical parameters and greater complete periodontal regeneration in single gingival recession treated with coronally advanced flap and SCTG combined with EMD compared with defects treated without EMD in animal models.

The ultimate goal of surgical treatment of GR should be outlined as not only root coverage percentage and complete root coverage of the recession defect (CRC) but also minimal probing depth and harmonious esthetics regarding profile of the gingival margin, soft-tissue texture, color, integration, and lack of visible scars [18]. Actually, esthetic failure might transpire even if CRC is ensured, but color match or tissue thickness is inadequate. All things considered, root coverage procedures should focus on total esthetic results to convey the outcomes more accurately. In this regard, root coverage esthetic score (RES) has been proposed and validated [18]. On the other hand, it is of utmost importance to take patient-centered outcomes and perceptions into account when evaluating the effectiveness of treatment modality that may represent true endpoints and better reflect the inherent value of treatment modalities. However, so far, patient-centered outcomes have been largely overlooked in clinical studies and limited number of randomized clinical trials (RCT) mentioned overall patient satisfaction and preference with regard to possible added benefit of EMD with the tunnel technique in gingival recession treatment.

The aim of this split-mouth and randomized controlled study was therefore to compare clinical efficacy of the modified coronally advanced tunnel technique (MCAT)+SCTG with or without EMD, in treatment of multiple RT1 and RT2. Moreover, another goal was to evaluate whether addition of EMD to SCTG reduces early morbidity associated with healing and enhances esthetic outcomes. Visual analog scales (VAS) and questionnaires were used to assess patient-reported outcomes and RES system for professional esthetic evaluation. The main outcome variable was percentage of root coverage and CRC at 6 months. Secondary outcome variables were reduction in GR, recession width (RW), gain in CAL, increase in gingival thickness (GT), increase in keratinized tissue width (KTW), and changes in RES values and patient-centered outcomes.

## Materials and methods

### Study design and subject population

**Fig. 1:**
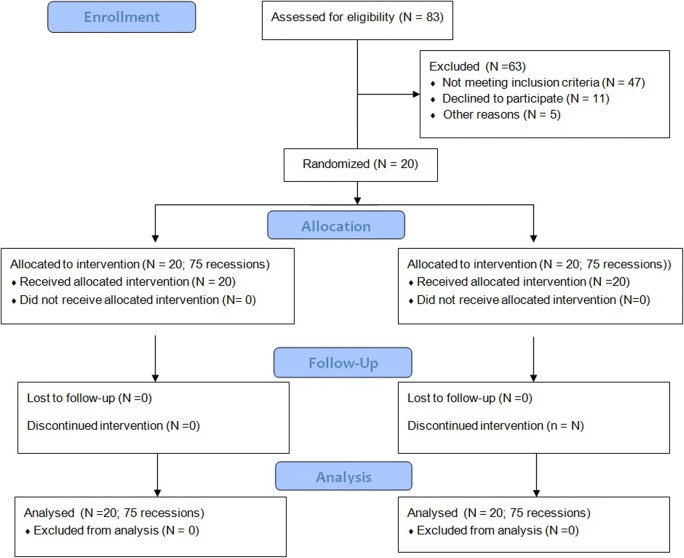
Consort diagram showing the study design

This study was designed as a single-center, double-blinded, split-mouth, randomized clinical trial. One side of the maxilla (or mandible) served as test, and the opposite side as control. The study was carried out in accordance with the Helsinki Declaration of 1975, as revised in Tokyo in 2004 after approval of the study design by the Bioethics Committee of Medical University of Warsaw (KB/208/2017). The study protocol was registered with ClinicalTrials.gov, Registration number: NCT03354104. The subject population was recruited among patients referred to the Department of Periodontology and Oral Mucosa Diseases of Medical University of Warsaw by their general dentists between January 2018 and June 2019 (Fig. 1). One examiner (TK) qualified patients into the study. Each patient signed an informed consent form before enrollment. Once the selected subjects agreed to participate in the study, they were instructed on how to use the roll technique with a soft toothbrush and provided with dental prophylaxis and polishing. Twenty patients (13 women and 7 men, aged 21–38; mean age 28.35 ± 4.51 years) were enrolled in the study, and one hundred fifty gingival recessions were treated. Defects were treated with MCAT in combination with SCTG either with (test, 75 defects) or without EMD (control, 75 defects).

### Inclusion and exclusion criteria

The inclusion criteria were established as follows: (1) at least two adjacent gingival recessions of recession type I and/or II at least 1-mm deep at homologous teeth in maxilla or mandible, (2) full-mouth plaque score (FMPS) < 20%, (3) full-mouth bleeding on probing (FMBOP) < 20%, (4) no active periodontal disease, (5) detectable cementoenamel junction (CEJ), (6) no caries lesions or restorations in the cervical area, (7) over 18 years of age, (8) no systemic diseases with compromised healing potential or infectious disease, (9) no use of medications affecting periodontal status, (10) no smoking, and (11) no pregnancy or lactation.

### Clinical measurements

Clinical parameters were assessed at baseline by the same experienced and calibrated examiner (SW) who was blinded with respect to the surgical procedures. A total of six non-study patients with at least two contralateral teeth with recessions were recruited for the calibration exercise. The designated examiner evaluated four teeth of each patient with an interval of 24 h between recordings. Calibration was accepted when ≥ 90% of the recordings could be reproduced within a difference of 1.0 mm, and an exact agreement was repeated in 75% of measurements. The following clinical parameters were evaluated for each gingival recession using a graded periodontal probe (UNC probe 15 mm, Hu-Friedy) and rounded off to the nearest 0.5 mm under local anesthesia: (1) gingival recession height (GR)—distance from CEJ to the most apical extension of gingival margin at mid-buccal point of the tooth, (2) RW—distance measured horizontally at CEJ level from one border of the recession to another, (3) probing pocket depth (PPD)—distance from the gingival margin to the bottom of the gingival sulcus at mid-buccal point of the tooth, (4) CAL—distance from CEJ to the bottom of the gingival sulcus at mid-buccal point of the tooth, (5) KTW—distance between the most apical point of the gingival margin and the muco-gingival junction (MGJ), (6) GT—measured at mid-buccal point of the tooth 3 mm apically from the gingival margin with the use of endodontic spreader 25 ISO (Poldent, Warsaw, Poland) and a silicon stopper put perpendicularly to the gingival surface until the alveolar bone or root surface was reached, (7) FMPS—the percentage of total surfaces (four aspects per tooth) that revealed the presence of plaque [19], and (8) FMBOP—assessed dichotomously at four points per tooth (mesio-buccal, mid-buccal, disto-buccal, mid-lingual) [20]. At 6 months, GR, RW, PPD, CAL, KTW, and GT were again recorded and the percentage of root coverage was measured.

### Sample size calculation

The sample size for paired continuous data was determined to be 18 subjects per treatment group, with the assumption that percentage of root coverage was the primary objective and based on the data that standard deviation (SD) of the differences in the paired measurements would not surpass 30% [21]. This would provide 80% power to disclose a true difference of 20% points between test and control. However, considering that some patients could be lost during follow-up, 20 patients were enrolled.

### Randomization and allocation concealment

Randomization was performed before surgical treatment by a statistician not involved in the study, who used a computerized random number generator. Allocation of treatment sites to test and control sites was concealed in sealed and opaque envelopes and was revealed to the surgeon immediately before the procedure. One envelope was opened to designate the surgical site located to the right to one of the two treatment modalities; subsequently, the surgical site to the left was treated in accordance with opposite treatment modality. No information on treatment allocation was provided to the patient.

### Surgical treatment

The surgical procedures were performed by one surgeon (BG) basically in accordance with the modified coronally advanced tunnel technique [9]. Both sides were treated during the same appointment, and the right side was always treated first. After local anesthesia with 4% articaine hydrochloride with adrenaline (1:100000) (Ubistesin Forte 1.7 ml, 3-M ESPE, Saint Paul, Minnesota, USA), the surgical area was prepared as a full-thickness flap with a small elevator up to MGJ and subsequently as a split-thickness flap above MGJ using the tunneling instruments. The papillary regions were detached in their buccal aspects with the periosteum. The adjacent papillae of the neighboring teeth were also involved in the preparation to ensure a coronal positioning of the tunnel flap. The exposed root surfaces were planed using designated curettes. In the next step, SCTG was harvested from the palate as epithelialized gingival graft [22]. After removing epithelium, the thickness of SCTG was less than 1 mm, and its width was around 4 mm. Graft length corresponded with the length of the recipient area in such a way that it overlapped evaluated gingival recessions. A hemostatic sponge was placed on the donor area and stabilized with cross-mattress non-resorbable sutures (Seralon 4/0 18 mm 3/8, Serag-Wiessner GmbH & Co. KG, Neila, Germany). Then, in the case of the test site, the root surfaces were conditioned with 24% EDTA (PrefGel, Straumann, Basel, Switzerland) for 2 min and washed with saline. EMD (Emdogain®, Straumann) was applied on the surface of the involved teeth before SCTG was put in place. For each site, one graft in one piece was used. SCTG was placed into the tunnel and stabilized at CEJ or 1 mm below the CEJ with resorbable sling sutures (PGA Resorba 6/0 11 mm 3/8, RESORBA Medical GmBH, Nürnberg, Germany). In the next step, the mobilized buccal flap was advanced coronally to fully cover SCTG and secured with 6/0 non-resorbable monofilament sling sutures (Seralon 6/0 12 mm 3/8, Serag-Wiessner GmbH & Co). On the control site, the recipient area was prepared in the same manner, but neither 24% EDTA nor EMD was used.

### Post-operative instructions and evaluation of morbidity

After the surgery, patients received 400 mg of ibuprofen and were asked to take the second dose 8 h later, as well as any additional tablets later on if required. The patients were provided with meticulous written post-operative instructions to avoid brushing, flossing, and chewing in the treated area for the period of 2 weeks. They were informed to rinse the mouth twice daily for 1 min using 0.2% chlorhexidine solution. On the day of intervention, patients were provided with a self-report questionnaire. It consisted of evaluation of pain and swelling in the operated areas using VAS. Each VAS consisted of a horizontal line, 10 cm (100 mm) in length, with a statement at each end representing one extreme of the variable being evaluated (the scale was anchored by “no pain or swelling” as score 0 and “worst imaginable pain or swelling” as score 100). The questionnaires were self-completed by the patients on the 1st, 2nd, 4th, 7th, and 14th day after surgery, who marked a line perpendicular to the VAS line at the point that represented intensity of their experiences. Check-up appointments were scheduled for 1, 2, and 4 weeks and later at 3 and 6 months. Each session consisted of reinforcement of oral hygiene instructions and supragingival plaque removal. At week 2, sutures were removed and patients were instructed in mechanical tooth cleaning of the operated sides using a soft toothbrush and the roll technique.

### Evaluation of esthetics and patient’s satisfaction

The esthetic outcome was evaluated 6 months post-operatively by independent second examiner (TK), who was blinded to the treatment assignment, according to RES [18]. The evaluation was based on comparing digital photographs taken at baseline and 6 months. Five variables were assessed and 0, 3, or 6 points were allocated to each variable: (1) gingival margin (GM)—0 point in case of failure of root coverage, 3 points in case of partial root coverage, and 6 points in case of complete root coverage (CRC); (2) marginal tissue contour (MTC) —0 point in case of irregular gingival margin and 1 point in case of proper marginal contour; (3) soft-tissue texture (STT) STT 0 point in case of scar formation and 1 point in case of absence of scar; (4) muco-gingival junction alignment STT 0 point in case of MGJ not aligned with the MGJ of adjacent teeth and 1 point in case of MGJ aligned with the MGJ of adjacent teeth; and (5) gingival color (GC) GC 0 point when color of tissue varies from gingival color at adjacent teeth and 1 point in case of normal color. The ideal esthetic score was 10.

At the 6-month follow-up examination, questionnaires were distributed to the patients for subjective evaluation of esthetics and overall satisfaction. Questions were designed in a dichotomous fashion (yes or no), and subsequently VAS was used to measure esthetic satisfaction.

### Statistical analysis

The clinical parameters, and esthetic and patient-centered outcomes were compared between two groups. Descriptive statistics were carried out using mean values, standard deviations (SD), frequencies, and percentages. Normality of distribution for quantitative variables was assessed using the Shapiro-Wilk test. All study quantitative variables were normally distributed. Therefore, the results of its variables were statistical analysis using the Student *t* test to compare means between two treatment groups. Comparison of fractions (percentages) was performed using Pearson’s chi-square test. To assess the validity of the treatment, the following calculations were executed: (1) recession reduction = GR0–GR6, (2) average root coverage (ARC) = GR0–GR6/GR0 × 100%, (3) CAL gain = CAL0–CAL 6, (4) KTW gain = KTW6–KTW0, and (5) GT gain = GT6–GT0. The two-way ANOVA was used to determine the significant difference between treatment groups for patients’ VAS-reported pain and swelling on the 1st, 2nd, 4th, and 7th day after the surgery. The analyses were performed with the R 3.2.3 software (R Core Team 2019). For all analyses, a significance level of 0.05 was assumed.

## Results

A total of 150 gingival recessions were treated (75 defects in the SCTG+EMD group and 75 defects in the SCTG group). Study teeth were maxillary incisors (28), canines (24), premolars (49), and first molars (13), as well as mandibular canines (10), premolars (21), and first molars (5). Fourteen subjects had recessions in the maxillary arch, and the other six presented defects in the mandibular arch. The majority of treated teeth were upper premolars (Table 1). Contralateral test and control defects were well balanced, and baseline data were homogeneous for all of the 20 involved subjects (Table 2). Healing was uneventful in all patients, all of whom completed scheduled appointments and the 6-month follow-up.

**Table 1:** Baseline characteristics for test and control groups

Variables	Test (*n* = 75)	Control (*n* = 75)
Tooth type (*n*)		
Incisors	14	14
Canines	17	17
Premolars	35	35
Molars	9	9
Tooth position (*n*)		
Maxillary teeth	56	58
Mandibular teeth	19	17
Class of GR according to Miller (*n*, %)		
Class I	60 (80)	64 (85.3)
Class II	2 (2.7)	1 (1.3)
Class III	13 (17.3)	10 (13.3)
Type of GR according to Cairo (*n*, %)		
RT1	65 (86.7)	66 (88)
RT2	10 (13.3)	9 (12)

*n* number of defects, *GR* gingival recession, *RT* recession type

**Table 2:** Clinical parameters (mean and standard deviation) at baseline and 6 months after surgery

	Baseline	6 months	*p*
GR SCTG+EMD (mm)	2.22 (1.00)	0.29 (0.77)	< 0.0001*
GR SCTG	2.16 (1.02)	0.24 (0.63)	< 0.0001*
*p*	0.7171	0.6724	
ARC SCTG+EMD (%)		87.49 (29.43)	
ARC SCTG		90.93 (23.20)	
*p*		0.4170	
GR red SCTG+EMD (mm)		1.91 (1.14)	
GR red SCTG		2.08 (1.50)	
*p*		0.4195	
RW SCTG+EMD (mm)	3.30 (1.38)	0.54 (1.34)	< 0.0001*
RW SCTG	3.25 (1.42)	0.56 (1.39)	< 0.0001*
*p*	0.7171	0.9277	
PPD SCTG+EMD (mm)	1.44 (0.58)	1.49 (0.62)	0.5781
PPD SCTG	1.43 (0.52)	1.60 (0.74)	0.0943
*p*	0.8822	0.3198	
CAL SCTG+EMD (mm)	3.56 (1.19)	1.30 (1.20)	< 0.0001*
CAL SCTG	3.25 (1.18)	1.29 (1.21)	< 0.0001*
*p*	0.1137	0.9630	
CAL gain SCTG+EMD (mm)		2.04 (1.40)	
CAL gain SCTG		1.79 (1.59)	
*p*		0.3001	
KTW SCTG+EMD (mm)	2.63 (1.42)	3.51 (1.33)	0.0001*
KTW SCTG	2.55 (1.27)	3.29 (1.31)	0.0309*
*p*	0.3190	0.3275	
KTW gain SCTG+EMD (mm)		0.94 (1.12)	
KTW gain SCTG		1.02 (1.27)	
*p*		0.6787	
GT SCTG+EMD (mm)	1.16 (0.34)	1.72 (0.43)	< 0.0001*
GT SCTG	1.18 (0.33)	1.78 (0.47)	< 0.0001*
*p*	0.1537	0.3930	
GT gain SCTG+EMD (mm)		0.58 (0.52)	
GT gain SCTG		0.68 (0.66)	
*p*		0.3161	

*GR* gingival recession height, *SCTG* subepithelial connective tissue graft, *EMD* Emdogain®, *ARC* average root coverage, *GR red* gingival recession reduction, *RW* gingival recession width, *PPD* probing pocket depth, *CAL* clinical attachment level, *KTW* keratinized tissue width, *GT* gingival thickness

*Statistically significant (*p* ≤ 0.05)

The clinical results at baseline and 6-month follow-up are depicted in Table 2. At 6 months, PPD values were not statistically different within and between groups. Significant decreases in GR, RW, and CAL were observed in both groups 6 months post-operatively compared with the baseline measurements, but no statistically significant differences between treatment groups were noted. In the test group, the mean recession height decreased significantly from 2.2 ± 1.0 (baseline) to 0.2 ± 0.7 mm (6 months), with a percentage of ARC of 87 ± 29 and a CRC in 65 out of 75 (86.7%) recession defects. In the control group, mean recession height decreased significantly from 2.1 ± 1.0 to 0.2 ± 0.6 mm, with a percentage of ARC of 90 ± 23 and a CRC in 64 out of 75 (85.3%) recession defects. Moreover, there was also a statistically significant CAL gain in the test and control groups (2.0 ± 1.4 and 1.7 ± 1.5 mm for the test and control groups, respectively). Both treatment resulted in a significant gain in KTW and GT on both sides: for KTW, from 2.6 ± 1.4 to 3.5 ± 1.3 mm on the SCTG+EMD side and from 2.5 ± 1.2 to 3.2 ± 1.3 mm on the SCTG side; for GT, from 1.1 ± 0.3 to 1.7 ± 0.4 on both sides. No significant differences with respect to CAL gain, WKT gain, and GT gain at 6 months between two treatment modalities were observed.

Both treatments showed high esthetic results. The root coverage esthetic score in the SCTG+EMD group was 9.2 ± 1.2, whereas in the SCTG group 8.7 ± 1.3 (*p* = 0.0103) (Table 3). In addition to average RES, there were also statistically significant differences in the three different parameters: marginal tissue contour, muco-gingival junction alignment, and gingival color, between two treatment modalities. All of the abovementioned were in favor of test sides. Keloid formation was not observed in any patient after 6 months.

**Table 3:** Evaluation of esthetic outcomes after 6 months (mean and standard deviation)

	GM	MTC	STT	MGJ	GC	RES
SCTG+EMD	5.48 (1.15)	0.94 (0.24)	0.89 (0.32)	0.99 (0.11)	0.96 (0.19)	9.25 (1.27)
SCTG	5.46 (1.16)	0.81 (0.40)	0.79 (0.41)	0.86 (0.35)	0.78 (0.42)	8.71 (1.37)
*p*	0.9415	0.0139*	0.1121	0.0021*	0.0005*	0.0103*

*SCTG* subepithelial connective tissue graft, *EMD* Emdogain®, *GM* gingival margin, *MTC* marginal tissue contour, *STT* soft-tissue texture, *MGJ* muco-gingival junction alignment, *GC* gingival color, *RES* root coverage esthetic score

*Statistically significant (*p* ≤ 0.05)

The values on the post-operative VAS are presented in Table 4. Patients reported significantly greater pain on the 1st, 2nd, 4th, and 7th day after the surgery on the control sides. Similar tendency was observed with respect to edema, but it did not reach statistical significance. Post-operative pain was reported by 13 patients in the test group and 17 patients in the control group, whereas postsurgical swelling by 17 and 19 subjects, respectively.

**Table 4:** Subject experience in term of post-operative morbidity

		Test (*n* = 75)					Control (*n* = 75)					*p*
		1st day	2nd day	4th day	7th day	14th day	1st day	2nd day	4th day	7th day	14th day	
Pain	*N* answering “yes” (%)	12 (60.00)	13 (65.00)	8 (40.00)	4 (20.00)	0 (0.00)	15 (75.00)	17 (85.00)	11 (55.00)	5 (25.00)	0 (0.00)	0.0165*
	VAS mean (SD)	3.54 (2.29)	3.47 (1.62)	2.63 (1.58)	1.70 (0.67)	0.00 (0.00)	3.63 (2.42)	3.69 (1.92)	2.95 (1.63)	2.13 (1.31)	0.00 (0.00)	0.0342*
Edema	*N* answering “yes” (%)	17 (85.00)	17 (85.00)	15 (75.00)	5 (25.00)	0 (0.00)	17 (85.00)	18 (90.00)	19 (95.00)	7 (35.00)	1 (5.00)	0.7549
	VAS (SD)	4.56 (2.26)	4.56 (2.25)	2.63 (1.01)	1.20 (0.45)	0 (0.00)	4.76 (2.13)	5.25 (1.73)	3.66 (1.82)	2.43 (2.59)	3.5 (0.00)	0.1137

*n*, *N* number, *VAS* visual analog scale, *SD* standard deviation

*Statistically significant (*p* ≤ 0.05)

Table 5 depicts patient-centered outcomes. No significant difference was detected between both groups with respect to the patients’ esthetic satisfaction describing gingival color, gingival contour, and gingival recession coverage, as measured by VAS values. When comparing SCTG+EMD and SCTG sides, VAS assessments for overall patient satisfaction were generally high with nearly identical mean values of 83.0 ± 12.5 for the test group and of 81.5 ± 13.5 for the control group. Almost all patients subjectively declared that they would decide again to go for the treatment performed and recommend it to another person.

**Table 5:** Results of patient questionnaire for evaluation of esthetics and overall satisfaction

Question	Test (*n* = 75)		Control (*n* = 75)		*p*
	*N* answering “yes” (%)	VAS mean (SD)	*N* answering “yes” (%)	VAS mean (SD)	
Gingival color		81.5 (13.03)		83.8 (12.37)	0.5792
Gingival contour		80.2 (14.89)		81.3 (13.33)	0.8238
Recession coverage		75.8 (15.04)		80.8 (13.80)	0.2790
“How satisfied are you with the results of the surgery?”	19 (95%)	83.0 (12.53)	18 (90%)	81.5 (13.47)	0.7172
“Would you decide again to go for the treatment performed?”	19 (95%)	84.2 (15.02)	19 (95%)	83.7 (15.33)	0.9155
“Would you recommend the treatment to another person?”	18 (90%)	80.8 (17.96)	18 (90%)	81.9 (17.36)	0.8475

*n*, *N* number, *VAS* visual analog scale, *SD* standard deviation

*Statistically significant (*p* ≤ 0.05)

**Fig. 2:**
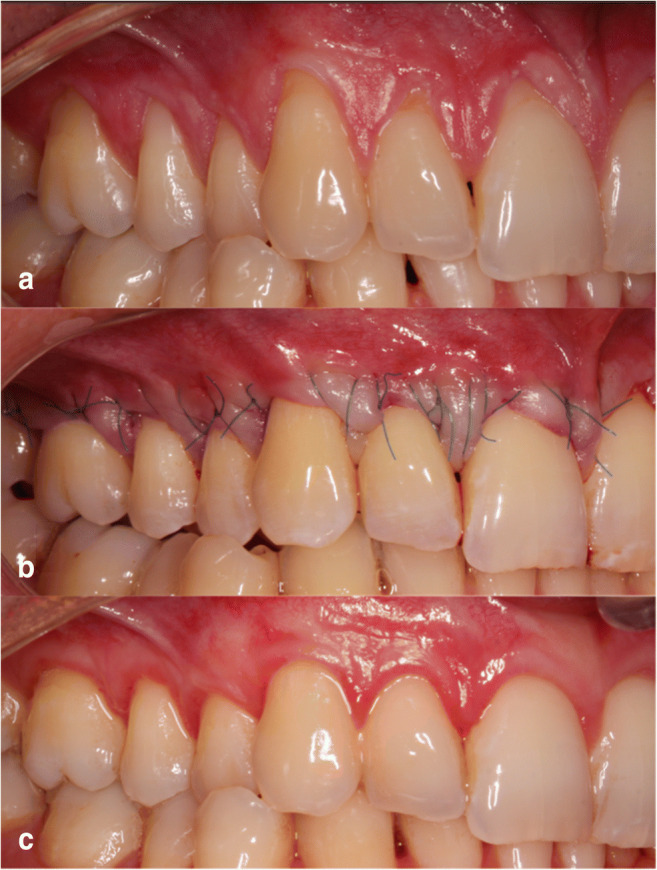
**a** Pre-operative view of gingival recessions on test side. **b** Immediate post-operative view. **c** 6 months post-operative view

**Fig. 3:**
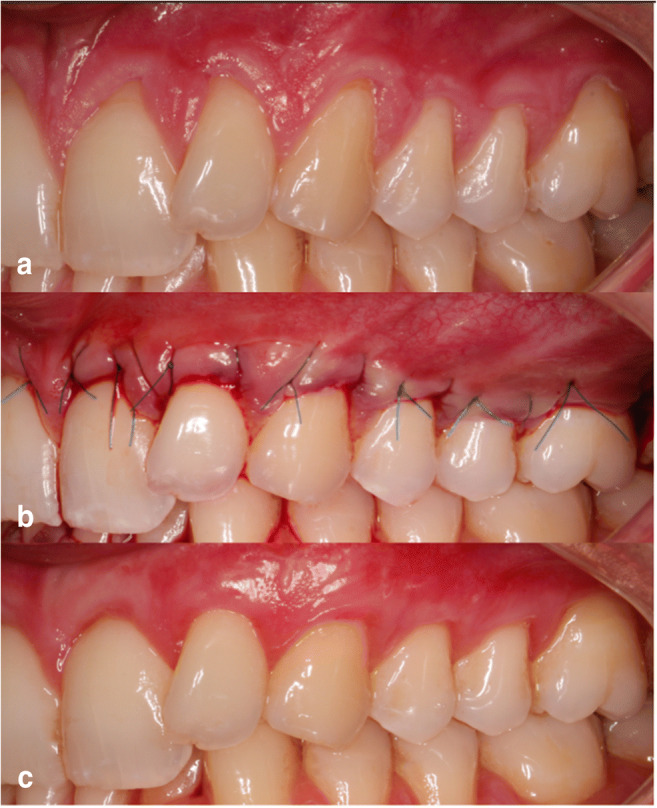
**a** Pre-operative view of gingival recessions in control side. **b** Immediate post-operative view. **c** 6 months post-operative view

Clinical outcomes in one patient are shown in Figs. 2 and 3.

## Discussion

A long-lasting debate in scientific community relates to the aspect of feasible influence of EMD on wound healing following gingival recession coverage. Based on the foundation of “biomimetics,” some authors recommended that EMD might be used as an adjunct to SCTG in surgical treatment of recessions with a view to enhance new periodontal attachment and facilitate post-operative healing. The objective of this study was to evaluate whether the combination of EMD with MCAF+SCTG would further improve treatment outcomes of RT1 and RT2 multiple defects. To the best of our knowledge, this is the second study of this kind, and the first one that evaluates esthetic and patient-centered outcomes.

Coming to the clinical findings of the present research, ARC and CRC were measured as primary outcome variables. At 6 months, ARC was 87.4% for SCTG+EMD-treated and 90.9% for SCTG-treated defects. CRC was observed in 86.7% (tests) and 85.3% (controls) of the cases. At 6 months, mean GR measured 0.29 mm in the test group and 0.24 mm in the control group. The abovementioned differences were not statistically significant. These outcomes compare well with those reported by other studies, where MCAT was used [10, 12, 23, 24], and are consistent with the conclusion of a recent systematic review and meta-analysis [8]. Consequently, the tunnel approach was found to be highly effective in treatment of multiple GR, exhibiting an overall root coverage of 87.9%, in addition to CRC of 57.5%. It was more sufficient in treating maxillary and Miller classes I and II GR. Furthermore, we observed equivalent improvements in other evaluated periodontal parameters for sites treated with or without EMD. KTW gain was 0.94 mm on sites where EMD was applied, and 1.02 mm on sites without EMD, whereas GT gain reached 0.58 and 0.68 mm, respectively. Apparently, the addition of EMD to SCTG in the MCAT technique did not seem to influence clinical outcomes. On the contrary, the additional use of EMD to SCTG in multiple recession coverage procedures with coronally advanced flap was associated with better root coverage and improved keratinized tissue [25]. Cheng et al. [26] performed meta-analysis and concluded that higher PPD reduction tends to occur when EMD was used additionally to SCTG. However, written evidence that took a comparative approach to efficacy of analyzed treatment modalities is rather scarce and only one study was found to compare clinical outcomes following treatment of Miller class I, II, or III recessions by means of MCAT+SCTG combined with and without EMD [12]. At 6 months, ARC measured 78 ± 26% in the test group and 77 ± 18% in the control group, while mean GR was 0.9 ± 1.3 mm and 1.0 ± 1.0 mm, respectively. No statistically significant differences were detected between the two groups, which is in agreement with our findings. The authors speculated that when MCAT is performed, root surfaces are not easily accessible and might be contaminated with blood, which, in turn, could alter the ability of EMD to precipitate on root surfaces and lessen its potential. It is therefore, at least from a clinical point of view, a reasonable hypothesis. Consistent with these findings, Aroca et al. [10, 23] reported ARC of 90% for the tunnel technique with CTG in treatment of multiple Miller classes I and II gingival recessions, and 83% in case of Miller class III gingival recessions after 12 months. The supplementary application of EMD to SCTG did not improve outcomes of multiple adjacent Miller III recessions coverage [10].

Another focus of this study was the evaluation of patient-centered outcomes and possible added benefit of EMD in this area. Written evidence is still inconsistent in this context. From the biological standpoint, it was previously reported that EMD could influence early wound healing by stimulation of migration, proliferation, and growth and metabolism of periodontal ligament cells and fibroblasts, as well as mesenchymal and microvascular cell differentiation and prevention of cell apoptosis [15]. All the abovementioned may possibly alleviate inflammation and pain symptoms. When EMD was topically applied in instrumented pockets, it improved the early healing of periodontal soft tissues [27]. At 1 week, the proportion of patients reporting a VAS score ≤ 20 was significantly greater for the EMD-treated quadrants than for controls (*p* = 0.002). However, little research has been carried out on perception of early post-operative discomfort and healing associated with MCAT. In the present study, the severity and duration of subjective pain and swelling were assessed by means of VAS scales evaluated directly after surgery, as well as in course of healing (up to suture removal). Although mean intensity of pain reported in both groups was rather low, a statistically significant reduction in pain severity was observed in the SCTG+EMD group, which is a clear clinical advantage. With regard to postsurgical edema, even though the results did not reach statistical significance, there was a trend toward less swelling being reported on test sites. No major adverse events were reported or observed. The maximum severity of pain and swelling was reported on 2nd day and was alleviated within 1 to 2 weeks after surgery. These findings are not in accordance with the results from the previously mentioned study. Stähli et al. [12] analyzed post-operative pain 2, 7, and 14 days after recession coverage. Mean VAS scores at the tooth site were 2.9, 2.9, and 1.1 for the SCTG+EMD group and 5.1, 3.5, and 1.6 for the SCTG group, without statistically significant differences. This discrepancy may be explained, at least in part, by different study design (the mentioned research did not adapt split-mouth fashion, and the distribution of GR varied from own study). The intensity of pain after MCAT was not as great as to exceed threshold. Moreover, in the cited research, inflammatory markers, such as interleukin IL-1β, IL-8, IL-10, and MMP-8, were measured at baseline, 2 days, and 1 week post-operatively, but no statistically significant difference between the two groups at any time and for any biomarker was found. The authors concluded that EMD did not influence immunological parameters related to wound healing following gingival recession surgery. On the other hand, our findings are remarkably similar to the results of a study by Mercado et al. [25], where the use of EMD as an adjunct to SCTG in the treatment of classes I and II Miller recessions resulted in significantly reduced pain on the 2nd, 7th, and 14th day after the surgery. In a very recent article, Lee et al. [28] reported no difference in the severity of early post-operative discomfort and wound-healing events between subjects who underwent periodontal surgery with and without EMD. However, duration of pain (*p* < 0.0001) and swelling (*p* = 0.019) was significantly shorter in patients who underwent treatment with EMD.

As far as we are aware, none of hitherto existing studies analyzed the additional benefit of EMD in combination with MCAT+SCTG for the treatment of multiple gingival recessions regarding professional appraisal, as well as patient-reported esthetic evaluation, which is a principal goal of periodontal plastic surgery. With the exception of dental hypersensitivity, esthetic concern constitutes the main reason for which patient requests recession treatment [29]. In light of this, the present study will contribute to literature. Objective measurements of the 6-month esthetic outcomes done by professional examiner using the RES score pinpointed a significant difference between both groups favoring SCTG+EMD (9.25 versus 8.71, *p* = 0.0103). As 60% of the RES value is affected by CRC, the remaining 40% is a result of other parameters. In the presented study, CRC did not differ significantly between two treatment modalities. Anyway, it should be kept in mind that percentage root coverage alone does not fully reflect esthetic results and that the other factors that contribute to both quantity and quality of soft tissue post-operatively should be taken into account when the outcomes of root coverage surgeries are evaluated. By the same token, other esthetic parameters, such as marginal tissue contour, MGJ alignment, and gingival color, were superior on SCTG+EMD sides, all of which allowed esthetically appealing integration of the grafts in a period of 6 months. In this context, the adjunctive use of EMD might be advocated for achieving enhanced esthetic outcomes. The rationale for these significant differences is open to speculation. One possible explanation is the characteristics related to EMD that stimulation of soft tissue healing and maturation might in turn accelerate the processes leading to improvement of proper profile of the gingival margin, MGJ return to baseline position, and better color matching. However, many different factors, such as flap thickness, tension, and excessive coronal displacement; discrepancies between MGJ in the operated areas and adjacent teeth; the dimension of SCTG; and the type of healing, might contribute to reported findings. Insufficient clinical studies comparing esthetic outcomes using RES in MCAT+SCTG with and without EMD prevent cross-referencing and drawing definitive conclusions on its beneficial effect. It is worthy to mention that the esthetic impact of EMD application in combination with SCTG+CAF in bilateral canine Miller class I or II gingival recessions was recently assessed [30]. Whereas there was no difference between two treatment modalities in terms of total RES and complete root coverage, the test group had significantly better results with regard to soft-tissue texture and MGJ alignment. Pietruska et al. [24] reported total RES of 8.36 for sides treated with MCAT+SCTG. However, MGJ and GC turned out to be higher in the case of root coverage with MCAT+collagen matrix, but these findings are not directly comparable to the data of the present experiment.

In terms of esthetic condition change and patients’ predilection for a specific procedure, no significant difference was detected between two treatment modalities. With respect to the patients’ esthetic satisfaction, VAS value evaluated in the questionnaires for the SCTG+EMD group was 83.0., whereas for SCTG group 81.5 (*p* = 0.7172), even though test sites were professionally assessed as having better outcomes, based on RES. Patients rated the achieved results as considerable improvement from baseline. A total of 19 patients declared that they were willing to undergo another periodontal surgery if necessary. Equally favorable results were also found for both treatment procedures with regard to patients’ perception of gingival color and marginal contour. Apparently, the differences in soft-tissue appearance between two treatment modalities were too subtle to determine subjective assessments of the esthetics, but any attempt to explain this outcome is speculative. These findings are slightly inferior to the results of a study by Zuhr et al. [31], who reported VAS values of 92.1 for tunnel-treated defects. In the aforecited study, both single and multiple recessions were treated. Quite similarly, there were no statistically significant differences with respect to patients’ satisfaction when EMD was added to SCTG in root coverage with coronally advanced flap [25]. All subjects in both groups reported that their expectations had been fulfilled 36 months after treatment. There was a trend toward more patients in the test group being prepared to have treatment again, if necessary (85.7% and 73.6%, respectively, *p* = 0.82). Within this frame of reference, our findings fill a gap in the knowledge in this field.

The present study is not without limitations. First, the potential effect of EDTA root conditioning on the reported outcomes cannot be ruled out, as it was performed only on test sites before EMD application. The influence of EDTA in this area remains controversial. Recent systematic review and meta-analysis pinpointed only limited evidence when assessing the capability of EDTA root conditioning with CAF+CTG but found this beneficial [32]. However, the impact of EDTA on MCAT has not been evaluated yet. In this regard, it is important to exercise caution when interpreting findings of the present study. It would be very worthwhile to analyze the efficacy of EDTA chemical root conditioning during MCAT treatment without EMD application in future studies. Different type of gingival recessions were evaluated, and due to limited number of RT2 defects, separate statistical analysis could not have been carried out. However, according to worldwide literature, this should not have a major influence on the observed findings, since recent studies did not support the importance of CAL as a predictor for root coverage in multiple recessions [33]. Furthermore, the efficacy of recession treatment depends on the type of included teeth, and soft-tissue augmentation at molar teeth poses a clinical challenge, yet the limited number of molars in both groups is very unlikely to influence the overall data. Another important limitation is the follow-up period of 6 months, as previous studies pinpointed that for esthetic assessment, the follow-up should not be shorter than 12 months [34]. A longer period of observation is possibly required to evaluate whether initial positive results of soft-tissue profile after EMD application are modified with time. Anyway, a long-term follow-up of the present patient population is intended. A split-mouth design was adapted in the present trial. To remove the impact of inter-individual variability from the estimates of treatment effect and to overcome the confounding effect that might arise from the healing of pretreated sites, both treatment strategies were implemented during the same session for each patient [35]. Be that as it may, without a control group consisting of no treatment, it was not plausible to analyze the impact of undergoing a surgical procedure on patients’ quality of life that may constitute another drawback of this research. All in all, the results of the present study should be confirmed in a larger group of subjects and longer observation time to elucidate and clarify our findings.

## Conclusions

Within the limitations of this 6-month study, the following conclusions can be drawn:

- modified coronally advanced tunnel technique with SCTG was very effective in treatment of multiple RT1 and RT2 defects;
- root coverage and outcome measures of evaluated periodontal parameters were not improved by adjunctive use of EMD;
- severity of early post-operative pain was reduced on sites treated with EMD;
- use of EMD as an adjunct enhanced professionally assessed esthetic outcomes (RES) 6 months post-operatively;
- subject-reported esthetic satisfaction with both test and control treatments was equivalent.

## References

[1] Kassab MM, Cohen RE (2003). The etiology and prevalence of gingival recession. J Am Dent Assoc.

[2] Jepsen S, Caton JG, Albandar JM, Bissada NF, Bouchard P, Cortellini P, Demirel K, de Sanctis M, Ercoli C, Fan J, Geurs NC, Hughes FJ, Jin L, Kantarci A, Lalla E, Madianos PN, Matthews D, McGuire MK, Mills MP, Preshaw PM, Reynolds MA, Sculean A, Susin C, West NX, Yamazaki K (2017). Periodontal manifestations of systemic diseases and developmental and acquired conditions: consensus report of workgroup 3 of the 2017 World Workshop on the Classification of Periodontal and Per-Implant Diseases and Conditions. J Clin Periodontol.

[3] Cairo F, Nieri M, Cincinelli S, Mervelt J, Pagliaro U (2011). The interproximal clinical attachment level to classify gingival recessions and predict root coverage outcomes: an explorative and reliability study. J Clin Periodontol.

[4] Miller PD (1985). A classification of marginal tissue recession. Int J Periodontics Rstorative Dent.

[5] Zabalegui I, Sicilia A, Cambra J, Gil J, Sanz M (1999). Treatment of multiple adjacent gingival recessions with the tunnel subepithelial connective tissue graft: a clinical report. Int J Periodontics Restorative Dent.

[6] Oates TW, Robinson M, Gunsolley JC (2003). Surgical therapies for treatment of gingival recession. A systematic review. Ann Periodontol.

[7] Santamaria MP, Neves FLDS, Silveira CA, Mathias IF, Fernandes-Dias SB, Jardini MAN, Tatakis DN (2017). Connective tissue graft and tunnel or trapezoidal flap for the treatment of single maxillary gingival recessions: a randomized clinical trial. J Clin Periodontol.

[8] Tavelli L, Barootchi S, Nguyen TVN, Tattan M, Ravidà A, Wang HL (2018). Efficacy of tunnel technique in the treatment of localized and multiple gingival recessions: a systematic review and meta-analysis. J Periodontol.

[9] Zuhr O, Fickl S, Wachtel H, Bolz W, Hürzeler MB (2007). Covering of gingival recessions with a modified microsurgical tunnel technique: case report. Int J Periodontics Restorative Dent.

[10] Aroca S, Keglevich T, Nikolidakis D, Gera I, Nagy K, Azzi R, Etienne D (2010). Treatment of class III multiple gingival recessions: a randomized-clinical trial. J Clin Periodontol.

[11] Sculean A, Cosgarea R, Stähli A, Katsaros C, Arweiler NB, Miron RJ, Deppe H (2016). Treatment of multiple adjacent maxillary Miller class I, II, and III gingival recessions with the modified coronally advanced tunnel, enamel matrix derivative, and subepithelial connective tissue graft: a report of 12 cases. Quintessence Int.

[12] Stähli A, Imber JC, Raptis E, Salvi GE, Eick S, Sculean A (2020). Effect of enamel matrix derivative on wound healing following gingival recession coverage using the modified coronally advanced tunnel and subepithelial connective tissue graft: a randomised, controlled, clinical study. Clin Oral Investig.

[13] McGuire MK, Cochran DL (2003). Evaluation of human recession defects treated with coronally advanced flaps and either enamel matrix derivative or connective tissue. Part 2: histological evaluation. J Periodontol.

[14] Rasperini G, Silvestri M, Schenk RK, Nevins ML (2000). Clinical and histological evaluation of human gingival recession treated with a subepithelial connective tissue graft and enamel matrix derivative (Emdogain): a case report. Int J Periodontics Restorative Dent.

[15] Miron RJ, Dard M, Weinreb M (2015). Enamel matrix derivative, inflammation and soft tissue wound healing. J Periodontal Res.

[16] Shirakata Y, Nakamura T, Shinohara Y, Nakamura-Hasegawa K, Hashiguchi C, Takeuchi N, Imafuji T, Sculean A, Noguchi K (2018). Split-mouth evaluation of connective tissue graft with or without enamel matrix derivative for the treatment of isolated gingival recession defects in dogs. Clin Oral Investig.

[17] Maymon-Gil T, Weinberg E, Nemcovsky C, Weinreb M (2016). Enamel matrix derivative promotes healing of a surgical wound in the rat oral mucosa. J Periodontol.

[18] Cairo F, Rotundo R, Miller PD, PiniPrato GP (2009). Root coverage esthetic score: a system to evaluate the esthetic outcome of the treatment of gingival recession through evaluation of clinical cases. J Periodontol.

[19] O'Leary TJ, Drake RB, Naylor JE (1972). The plaque control record. J Periodontol.

[20] Ainamo J, Bay I (1975). Problems and proposals for recording gingivitis and plaque. Int Dent J.

[21] Julious SA, Campbell MJ (1998). Sample size calculations for paired or matched ordinal data. Stat Med.

[22] Zucchelli G, Mele M, Stefanini M, Mazzotti C, Marzadori M, Montebugnoli L, de Sanctis M (2010). Patient morbidity and root coverage outcome after subepithelial connective tissue and de-epithelialized grafts: a comparative randomized controlled clinical trial. J Clin Periodontol.

[23] Aroca S, Molnar B, Windisch P, Gera I, Salvi GE, Nikolidakis S, Sculean A (2013). Treatment of multiple adjacent Millar class I and II gingival recessions with a modified coronally advanced tunnel (MCAT) technique and a collagen matrix or palatal connective tissue graft: a randomized, controlled clinical trial. J Clin Periodontol.

[24] Pietruska M, Skurska A, Podlewski Ł, Milewski R, Pietruski J (2019). Clinical evaluation of Miller I and II recessions treatment with the use of modified coronally advanced tunnel technique with either collagen matrix or subepithelial connective tissue graft: a randomized clinical study. J Clin Periodontol.

[25] Mercado F, Hamlet S, Ivanovski S (2019). A 3-year prospective clinical and patient-centered trial on subepithelial connective tissue graft with or without enamel matrix derivative in class I-II Miller recessions. J Periodontol.

[26] Cheng GL, Fu E, Tu YK, Shen EC, Chiu HC, Huang RY, Yuh DY, Chiang CY (2015). Root coverage by coronally advanced flap with connective tissue graft and/or enamel matrix derivative: a meta-analysis. J Periodontal Res.

[27] Wennström JL, Lindhe J (2002). Some effects of enamel matrix proteins on wound healing in the dento-gingival region. J Clin Periodontol.

[28] Lee JH, Park YS, Kim YT, Jeong SN (2020). Assessment of early discomfort and wound healing outcomes after periodontal surgery with and without enamel matrix derivative: an observational retrospective case-control study. Clin Oral Investig.

[29] Nieri M, Pini Prato GP, Giani M, Magnani N, Pagliaro U, Rotundo R (2013). Patient perceptions of buccal gingival recessions and request for treatment. J Clin Periodontol.

[30] Aydinyurt HS, Tekin Y, Ertugrul AS (2019). The effect of enamel matrix derivatives on root coverage: a 12-month follow-up of a randomized clinical trial. Braz Oral Res.

[31] Zuhr O, Bäumer D, Hürzeler M (2014). The addition of soft tissue replacement grafts in plastic periodontal and implant surgery: critical elements in design and execution. J Clin Periodontol.

[32] Barootchi S, Tavelli L, Ravidà A, Wang CW, Wang HL (2018). Effect of EDTA root conditioning on the outcome of coronally advanced flap with connective tissue graft: a systematic review and meta-analysis. Clin Oral Investig.

[33] Aroca S, Antoine B, Clementini M, Renouard F, de Sanctis M (2018). Treatment of class III multiple gingival recessions: prognostic factors for achieving a complete root coverage. J Clin Periodontol.

[34] Kerner S, Sarfati A, Katsahian S, Jaumet V, Micheau C, Mora F, Monnet-Corti V, Bouchard P (2009). Qualitative cosmetic evaluation after root-coverage procedures. J Periodontol.

[35] Lesaffre E, Philstrom B, Needleman I, Worthington H (2009). The design and analysis of split-mouth studies: what statisticians and clinicians should know. Stat Med.

